# Mutant Analysis in Arabidopsis Provides Insight into the Molecular Mode of Action of the Auxinic Herbicide Dicamba

**DOI:** 10.1371/journal.pone.0017245

**Published:** 2011-03-08

**Authors:** Cynthia Gleason, Rhonda C. Foley, Karam B. Singh

**Affiliations:** 1 CSIRO Plant Industry, Centre for Environment and Life Sciences, Wembley, Western Australia, Australia; 2 The University of Western Australia Institute of Agriculture, The University of Western Australia, Crawley, Western Australia, Australia; Instituto de Biología Molecular y Celular de Plantas, Spain

## Abstract

Herbicides that mimic the natural auxin indole-3-acetic acid are widely used in weed control. One common auxin-like herbicide is dicamba, but despite its wide use, plant gene responses to dicamba have never been extensively studied. To further understand dicamba's mode of action, we utilized Arabidopsis auxin-insensitive mutants and compared their sensitivity to dicamba and the widely-studied auxinic herbicide 2,4-dichlorophenoxyacetic acid (2,4-D). The mutant *axr4-2*, which has disrupted auxin transport into cells, was resistant to 2,4-D but susceptible to dicamba. By comparing dicamba resistance in auxin signalling F-box receptor mutants (*tir1-1*, *afb1*, *afb2*, *afb3*, and *afb5*), only *tir1-1* and *afb5* were resistant to dicamba, and this resistance was additive in the double *tir1-1/afb5* mutant. Interestingly, *tir1-1* but not *afb5* was resistant to 2,4-D. Whole genome analysis of dicamba-induced gene expression showed that 10 hours after application, dicamba stimulated many stress-responsive and signalling genes, including those involved in biosynthesis or signalling of auxin, ethylene, and abscisic acid (ABA), with TIR1 and AFB5 required for the dicamba-responsiveness of some genes. Research into dicamba-regulated gene expression and the selectivity of auxin receptors has provided molecular insight into dicamba-regulated signalling and could help in the development of novel herbicide resistance in crop plants.

## Introduction

Auxinic herbicides are synthetic auxins that have been effectively used in agriculture to control broadleaf weeds for over 60 years [1]. Synthetic auxins act as mimics of natural auxin, and they can be categorized into different classes based on the position of their carboxycylic acid moieties on their aromatic rings. The classes include phenoxyalkanoic acids (e.g. 2,4-D), benzoic acids (e.g. dicamba), and the pyridine-carboxylic acids (e.g. picloram) [2]. In response to an auxinic herbicide, the plant develops abnormalities such as leaf epinasty, leaf abscission, and growth inhibition of the root and shoots [1], [2], [3], [4]. Overall, the effects of auxinic herbicides can be divided into three consecutive phases in the plant: first, stimulation of abnormal growth and gene expression; second, inhibition of growth and physiological responses, such as stomatal closure; and third, senescence and cell death [3]. It is during the stimulation phase that auxinic herbicides cause a rapid increase in ethylene production and an increase in abscisic acid (ABA) biosynthesis [5], [6], [7]. The increased ABA levels inhibit plant growth by closing the stomata, which subsequently limits carbon dioxide assimilation and leads to the accumulation of hydrogen peroxide in the herbicide-treated plants (second phase effects) [7]. This accumulation of reactive oxygen species is likely a key factor contributing to the tissue damage and cell death associated with herbicide treatment (third phase effects) [3].

The natural auxin indole-3-acetic acid (IAA) enters the cell through auxin-influx carriers and rapidly controls auxin-responsive gene expression by regulating the degradation of Aux/IAA repressor proteins. Aux/IAA proteins are negative regulators of auxin-responsive genes [8]. Auxin binds to an F-box protein called TIR1, a subunit of the SCF^TIR1^ (Skp-Cullin-F-box) ubiquitin ligase protein complex. TIR1 directly binds auxin, and this binding allows TIR1 to associate with Aux/IAA proteins. The Aux/IAA repressor proteins are ubiquitinated by the SCF^TIR1^ complex [9], [10] and degraded by the 26S proteasome. The removal of the Aux/IAA proteins relieves the repression of auxin-responsive genes [8], [11].

Arabidopsis has a number of TIR1-like F-box proteins, with AFB1, AFB2 and AFB3 showing the closest homology to TIR1 [12], [13]. Like TIR1, the AFB1, AFB2, and AFB3 proteins can bind to IAA, but their role in auxin signalling is not clear. At least one AFB protein, AFB5, plays a role in synthetic auxin selectivity. A mutant in AFB5 was resistant to the auxinic herbicide picloram, suggesting that plant responses to this herbicide was mediated, at least in part, through the SCF^AFB5^ pathway [14]. Because synthetic auxins, such as 2,4-D, differ in their overall aromatic ring size from natural auxin, the potential chemical selectivity of AFBs may be based on the structure or size of their auxinic-binding pockets [15]. Understanding the relationship between auxin receptors and synthetic auxins may be critical for dissecting their modes of action and for the development of specific herbicide resistance in crop plants.

In this paper we wanted to gain a better understanding of the global molecular effects of dicamba (2-methoxy-3,6-dichlorobenzoic acid) on plants. Dicamba has low toxicity to animals and is widely-used to control broadleaf weeds [16]. In a 2001 study, dicamba was the 7^th^ most-used home and garden herbicide and was ranked the 24^th^ in a list of the most-commonly used agricultural herbicides [17]. With the emergence of glyphosate resistant weeds, alternative herbicides like dicamba will likely gain increased usage. Moreover, expression of a bacterial dicamba monooxygenase gene was shown to confer dicamba resistance to transgenic plants [16], which is likely to increase its popularity in agriculture. While resistance to dicamba has been observed in some weed species [18], [19], [20], the underlying mechanisms of these resistances are unknown, and effects of dicamba on plants at the molecular level have not been extensively characterized. For our study we used the model plant Arabidopsis, which has a wealth of molecular genetic and genomic resources that we could exploit [8], [21], [22]. We utilized whole genome microarray analysis and identified functional categories of genes affected by this herbicide that help explain dicamba's mode of action. We also examined several auxin insensitive mutants and discovered differential responses to dicamba and 2,4-D. Based on this mutant work, we hypothesize that dicamba and 2,4-D may have different requirements for entry into the cell and for F-box receptors that bind to the synthetic auxins.

## Materials and Methods

### Plant material, treatments, and RNA extraction

Arabidopsis seeds were surface sterilized for 15 minutes in 70% ethanol and rinsed with 1 ml 100% ethanol. The seeds were stratified for 3 d at 4°C and grown on Murashige and Skoog agar plates supplemented with 3% sucrose and 0.8% agar, under 16-h-light/8-h-dark cycle at 22°C. The *GSTF8*::LUC plants were transgenic Columbia containing a 791 bp version of the *GSTF8* promoter fused to the luciferase reporter gene [23], [24]. Homozygous seeds for *tir1-1* (CS3798) [25], *axr4-2* (CS8019) [26], *afb1-3* (SALK_070172.53.50.x), and *afb3-4* (SALK_068787C) and heterozygous seeds for *afb5* (SALK_110643.30.55.x) and *afb2* (SALK_137151.27.45.x) were provided by the Arabidopsis Biological Resource Center. Mutants alleles have been previously described for the SALK T-DNA insertions *afb1-3* and *afb3-4* [13]. Homozygous plants for *afb5* and *afb2* were selected on MS-media with Kanamycin (50 µg/ml) and further confirmed by PCR with gene specific primers: *afb5* 5′- GTTGGATCTACCCTCTACCGC-3′, 5′-GTGGCAATTGAGTATGATGGG-3′; *afb2* 5′- TCAACGGTCAAGATCCATCTC-3′ and 5′- CTGCAATTAGCGGCAATAGAG -3′, and a primer within the T-DNA: LBb1.3 ATTTTGCCGATTTCGGAAC. Loss of transcript in the T-DNA lines was confirmed by loss of amplicon from cDNA template with primers: *afb5* 5′- TGTGGAGCTACATCGTCTGC-3′ and 5′-GGAAGATACTCCGGCATCAA-3 and *afb2* 5′- TCTGGTTCCTTTGCTTTGCT-3′ and 5′-TCGGAATCTGGGTCATTCTC -3′. The *tir1-1/afb5* double mutant was generated through a cross of the two single mutant lines, and homozygous lines were generated and confirmed for *tir1-1* using CAPS marker [27] and for *afb5*, using primers described above.

The 7 mM dicamba treatment was made by dissolving 22.8 mg Cadence (700 g/kg dicamba, Syngenta; mw  =  325 g/mole) in 10 ml water. For the gene expression experiments, 4 day old seedlings were flooded with 10 ml of a 7 mM dicamba treatment (Cadence, Syngenta) or water (mock) for 40 minutes. At the specified timepoints after the initial exposure to the treatments, the plants were collected and quick frozen in liquid nitrogen. For all experiments, total RNA was extracted from whole seedlings using the Qiagen RNeasy Mini Kit, following the manufacturer's instructions.

### Microarray and data analysis

The cDNA preparation, labelling, and Affymetrix ATH-121501 GeneChip® hybridization was carried out by AGRF (Australian Genome Research Facility, http://www.agrf.org.au) using 15 ug of total RNA and three biological replicates. The raw data files underwent background correction, normalization, probe specific correction, and summary value computation using the Robust Multichip Average (RMA) method in the Bioconductor package. To find the differentially expressed genes we utilized the limma package for R to calculate the Log_2_ fold change, the moderate t-statistic, B value, and the adjusted p value (Benjamini–Hochberg *FDR*) for each array probe [28]. Functional categorization was carried out using the Arabidopsis Classification Superviewer at http://bar.utoronto.ca/ntools/cgi-bin/ntools_classification_superviewer.cgi [29]. It should be noted that with SuperViewer genes can belong to more than one group of classification. Microarray data from this article were submitted to the public NCBI Gene Expression Omnibus database (GEO) accession GSE24052, The microarray data is MIAME compliant.

### Root growth assays

Surface sterilized Arabidopsis seeds were sown on MS-media supplemented with increasing concentrations of herbicides. A concentrated dicamba stock (7 mM) was diluted in water. The concentrated 2,4-D stock (1 mM) was made in DMSO and then diluted in water. Control plates had appropriate amounts of DMSO or water without herbicide. Ten seeds of Col-0, *tir1-1*, and *axr4-2* were sown on each plate. The plants were stratified for 3 days at 4°C and then placed in a 21°C growth room with a 16 hour light/8 hour dark cycle. Eight days after seeds were placed in the growth room, the plants were removed from the agar medium and the length of primary root was measured. There were 2 replicate plates of at least 10 plants for each herbicide concentration tested and the experiment was repeated at least twice.

### Plant-herbicide resistance tests

Surface sterilized seeds were stratified and germinated on MS-media. After 1.5 weeks of growth in a 21°C growth room, the plants were transplanted into soil and allowed to grow an additional 3 weeks with regular watering. 20 mls–40 mls of dicamba treatment were applied to each pot. Three days later, the total above ground fresh weight was measured. Each 6.5×6.5 cm pot contained 5 plants. A total of 20 plants each of Col-0 and *tir1-1* were measured for each concentration of dicamba treatment. The experiment was repeated twice with similar results.

### Gene expression analysis

cDNA was generated from 1 µg total RNA using Invitrogen's SuperScript II Reverse Transcriptase following manufacturer's instructions. The cDNA was used as the template in real-time quantitative PCR; the qRT-PCR conditions and analysis were similar to those used in the study of Gao and associates [30]. Fragments of interest were amplified by the Biorad iCycler Real Time PCR machine using Biorad SYBR green as the fluorescent dye. Relative gene expression was derived from using 2^–ΔCT^, where C_T_ represents C_T_ of the gene of interest minus C_T_ of the reference gene cyclophilin (*ATCYP5*). Where required, the significance of differences between relative gene expressions was analysed by two-way ANOVA. Each experiment was done in duplicate with three biological replicates. For verification of the Affymetrix microarray results, cDNA was synthesized from RNA isolated for the microarray experiment (above) and from an independent experiment set-up in an identical manner. Gene specific primers for the qRT-PCR are listed in Table S3.

## Results

### Transcriptome analysis of genes affected by dicamba treatment

In order to determine how dicamba is affecting specific gene expression and global signalling pathways, we anaysed transcripts using an Affymetrix Arabidopsis ATH-121501 array. To establish the dicamba concentration and time after treatment to collect tissue for transcriptional analysis, we utilized plants carrying a stress responsive gene, *GSTF8*, fused to a luciferase reporter, which acted as a marker of maximal transcriptional activity in the plant after dicamba treatment. It was previously reported that 7 mM dicamba can strongly induce the *GSTF8* promoter in 4 day old seedlings [31]. This dicamba concentration also falls within the range used in agricultural practices to kill weeds (0.28 kg/ha and 0.56 kg/ha, applied at approximately 180 L/ha) [16], and thus, would give an insight into gene expression in herbicide-treated plants in field situations. Using 7 mM dicamba, we found that the *GSTF8* promoter activity peaked 7–10 hours after treatment (data not shown), and thus, we analysed gene expression using the array on plant tissue collected 10 hours after a 7 mM dicamba treatment. At this ten hours after treatment, there would be maximal transcriptional activity, as indicated by the peak in *GSTF8* promoter activity, and, it would provide insights into the prolonged effects of dicamba on plant gene expression and complement the transcriptional analyses performed with auxinic herbicides at early timepoints [32], [33], [34]. Genes with ≥2-fold change between the mock and dicamba treated seedlings were extracted for further analysis. With these criteria, 1192 probe identifiers were up-regulated and 1003 were down-regulated in the dicamba treated plants.

**Figure 1:**
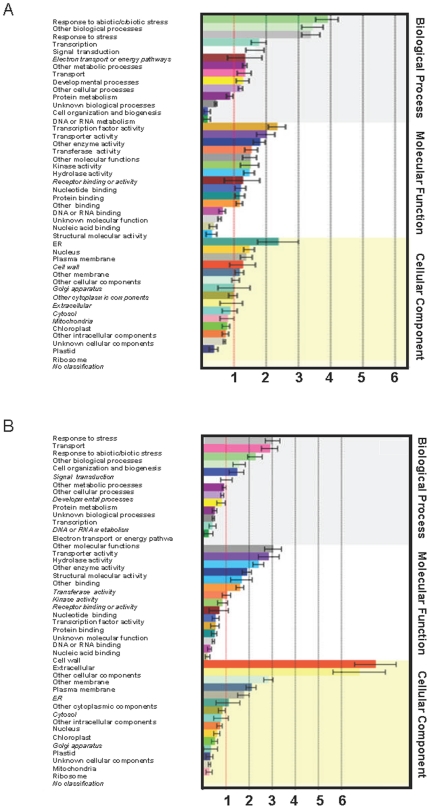
Functional annotations of genes regulated by dicamba treatment. Functional classification of genes regulated by dicamba based on the Gene Ontology (GO) database using the Arabidopsis Classification SuperViewer [29], A, Genes induced B, Genes repressed. Four day old seedlings were collected 10 hours after a 7 mM dicamba treatment for the array, with 3 biological replicates. All genes included in the tables had fold change ≥3 and an adjusted p≤0.05. The values are a normed frequency, which is calculated as follows: (Number_in_Class_input_set_/Number_Classified_input_set_)/(Number_in_Class_reference_set_/Number_Classified_reference_set_) (error bars  =  bootstrap StdDev). Genes with a p-value of the hypergeometric distribution >0.5 are in italics. Full records of differentially expressed genes are listed in Tables S1 and S2.

Because this was a large data set, for further analysis, genes with greater than 3-fold differential expression were functionally categorized (Fig. 1). This consisted of 550 induced genes and 396 repressed genes (Tables S1 and S2). For most of the categories, such as “Responses to abiotic/biotic stress,” “Responses to stress,” “Transcription,” and “Signal transduction,” there were more genes induced by dicamba than repressed (Fig 1A).

To confirm the changes in gene expression observed in the microarray experiment, we performed quantitative real time PCR (qRT-PCR) for a select number of genes. Expression patterns of several genes that are involved in the biosynthesis or signalling of ethylene, ABA, or auxin were studied, and qRT-PCR results confirmed the changes in gene expression by dicamba as determined by the microarray (Table 1).

**Table 1:** Quality control of microarray experiments.

Pathway	AGI	Gene Name	Microarray FC	qRT-PCR FC
ethylene	At4g08040	*ACS11*	3.4	4.4
ABA	At3g14440	*NCED3*	3.8	3.6
auxin	At2g23170	*GH3.3*	19.2	28.6
auxin	At1g15580	*IAA5*	7.8	10.2
auxin	At4g14560	*IAA1*	6.2	9.3

The fold change (FC) of genes that were differentially expressed during the microarray analysis after dicamba treatment were confirmed by qRT-PCR. Each qRT-PCR experiment was done twice with the average of the fold change reported.

### Genes up-regulated by dicamba

“Response to abiotic or biotic stresses,” “Other biological processes,” and “Response to stress” comprised the largest categories of dicamba-induced genes (Fig. 1A). Stress responsive genes included five glutathione S-transferases of the tau class and 12 cytochrome p450s, suggesting a role of p450s and GSTs in the dicamba detoxification process. Of the genes induced 3-fold or more by dicamba, a significant number were also involved in transcription, for example those encoding DREB2A and WRKY33, or were genes involved in signalling, including genes encoding calcium-binding proteins and kinases (Table S1).

The proposed model for auxinic herbicides predicts that the high levels of auxin induce ethylene biosynthesis which is followed by ABA biosynthesis [3]. Consequently, we analysed dicamba-induced genes to indentify those involved in auxin, ethylene, and ABA biosynthesis and/or signalling pathways.

Several auxin responsive genes were up-regulated upon dicamba treatment, and these included six IAA transcription factors and 7 auxin-responsive genes. The Aux/IAAs are known to help repress the expression of auxin-induced genes and ensure that the auxin response is transient [35], and the fact that they are induced 10 hours after dicamba treatment suggests a sustained auxin response in the plant. Some of the most rapidly induced genes by IAA encode indole-3-acetic acid amino synthetases (GH3s) that can conjugate IAA to help maintain auxin homeostasis (Kelley and Riechers 2007, Staswick *et al.* 2005); there are several GH3 family members in Arabidopsis, of which four (*GH3.1*, *GH3.3*, *GH3.5* and *GH3.6*) were induced over 3-fold by the 7 mM dicamba treatment (Table S1).

Auxin can increase plant ethylene concentrations by inducing the synthesis of 1-aminocyclopropane-1-carboxylic acid (ACC) synthase, a key step in ethylene biosynthesis in which S-adenosylmethionine is converted to ACC [36]. We discovered that 3 ACC synthase family members, including *ACS11* and the auxin-inducible *ACS8*, were induced by dicamba treatment. In addition, an ACC oxidase, which catalyses the final step in ethylene biosynthesis, was also up-regulated in the dicamba-treated plants.

The plastid enzyme 9-cis-epoxycarotenoid dioxygenase (NCED) catalyses a key regulatory step in ABA biosynthesis [37]. Our genome expression analysis showed that two NCED family members, *NCED3* and *NCED5*, were up-regulated after dicamba exposure. Abscisic aldehyde oxidase 3 (At2g27150), an enzyme that catalyses the final step of ABA biosynthesis, was also induced upon dicamba treatment. In addition to the biosynthetic genes, at least 10 ABA responsive genes were found to be up-regulated by dicamba over 3 fold. Reducing the stringency of the fold change cut-off in our analysis to 2.5 fold induction revealed that a zeaxanthin epoxidase gene (ABA1), which catalyses the first step of ABA biosynthesis, was also induced by dicamba.

### Genes repressed by dicamba

In most of the categories analysed, there were fewer genes repressed by dicamba than induced (Tables S1 and S2). The major exceptions being genes falling into the categories “Cell wall” and “Extracellular” (Fig. 1B and Table S2).

A significant number of cell wall genes were specifically down-regulated after dicamba exposure, suggesting that dicamba may be inhibiting cell wall biosynthesis (Fig. 1B). Our data also showed that 24 predicted peroxidases were specifically repressed by dicamba (compared with none that are induced). Fifteen encoded putative peroxidases and the rest were class III peroxidases [38]. Peroxidases have many physiological functions, including cell wall lignification and IAA catabolism [39].

The transcriptome results also showed that transport associated genes, particularly lipid transport proteins (LTPs) and the water transport proteins [plasma membrane intrinsic proteins (PIPs) and tonoplast intrinsic proteins (TIPs)], were all down-regulated by dicamba.

### Comparison of dicamba resistance in ethylene, ABA, and auxin insensitive mutants

**Figure 2:**
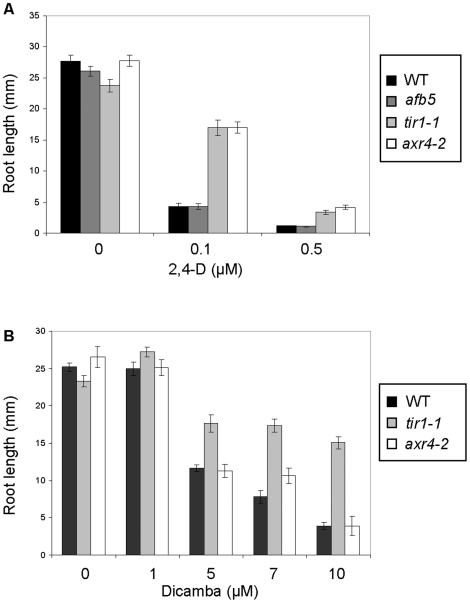
Differential effects of 2,4-D and dicamba on the root growth of WT and auxin mutant plants. The data represents primary root length measured after 8 days on media with various concentrations of herbicide. Measurements were taken on at least 10 seedlings per plate and at least 2 plates per treatment. Error bar indicated standard error (sem). A, Effects of 2,4-D (0, 0.1 µM, and 0.5 µM) on root length compared between WT, *axr4-2*, *afb5*, and *tir1-1* plants. B, The effects of dicamba (0, 1, 5, 7 and 10 µM) on the root lengths of WT, *axr4-2*, and *tir1-1* plants.

Whole genome expression analysis showed that auxin, ethylene and ABA biosynthesis and/or signalling genes were induced by dicamba, suggesting that these hormones may be critical for dicamba sensitivity. Therefore, we tested several phytohormone-insensitive mutants for enhanced dicamba resistance. One effect of both natural and synthetic auxin treatment is a reduction in root growth, and thus, effects on root growth was used as a criteria for measuring altered dicamba sensitivity in Arabidopsis mutant backgrounds. In order to establish the concentration at which the herbicides affect root growth, we measured root lengths of Columbia (wildtype, WT) plants grown on increasing concentrations of either 2,4-D or dicamba. 2,4-D affected growth of WT roots at concentrations of 0.1 and 0.5 µM, which is similar to previously published reports, and illustrates that we can replicate the effects of synthetic auxin on plant growth under our laboratory growth conditions [12], [26] (Fig. 2A). Dicamba concentrations between 0.1 and 1 µM did not significantly affect WT root growth, but at concentrations of 5 µM and 7 µM, the WT roots were 46% and 31%, respectively, the length of the untreated control (Fig. 2B).

**Figure 3:**
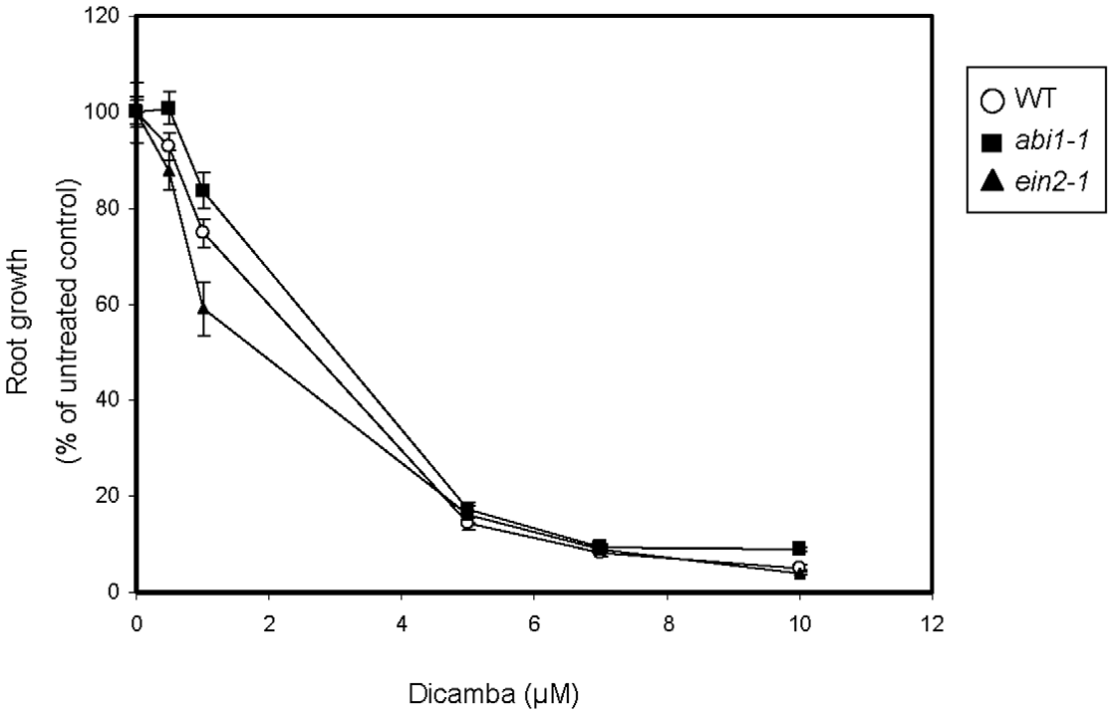
Effects on WT, *abi1-1* and *ein2-1* root growth as dicamba concentrations increase. Root growth measured after 8 days on MS-media containing 0, 0.5, 1, 5, 7 or 10 µM dicamba and transformed to a percentage of the average root length of the untreated control for each genotype. Measurements were taken on at least 20 seedlings of each line. (Error bars  =  standard error)

Because the transcript analysis suggested that ethylene and ABA –mediated pathways in the plant may be affected by dicamba, we first tested ABA-insensitive Arabidopsis *abi1-1* [40], and the ethylene insensitive *ein2-1* [41] for enhanced dicamba resistance. However, both mutants showed susceptibility to dicamba similar to WT plants (Fig. 3).

We next assayed the auxin insensitive mutants *axr4-2* and *tir1-1* for dicamba resistance. We initially chose these mutants because they are affected in different aspects of auxin signalling and either play a role in auxin transport (AXR4) or auxin binding (TIR1). The *tir1-1* plants were partially resistant to 5, 7, and 10 µM dicamba concentrations. There was no *tir1-1* resistance to dicamba at concentrations of 20 µM and above (data not shown). Meanwhile *axr4-2* showed sensitivity to dicamba similar to WT plants (Fig. 2B). As has been previously described, both *axr4-2* and *tir1-1* exhibited partial resistance to 2,4-D (Fig. 2A) [25], [42]. In summary, we have shown that *tir1-1* has at least partial resistance to both dicamba and 2,4-D, but *axr4-2* exhibited differential resistance between the two auxinic herbicides.

**Figure 4:**
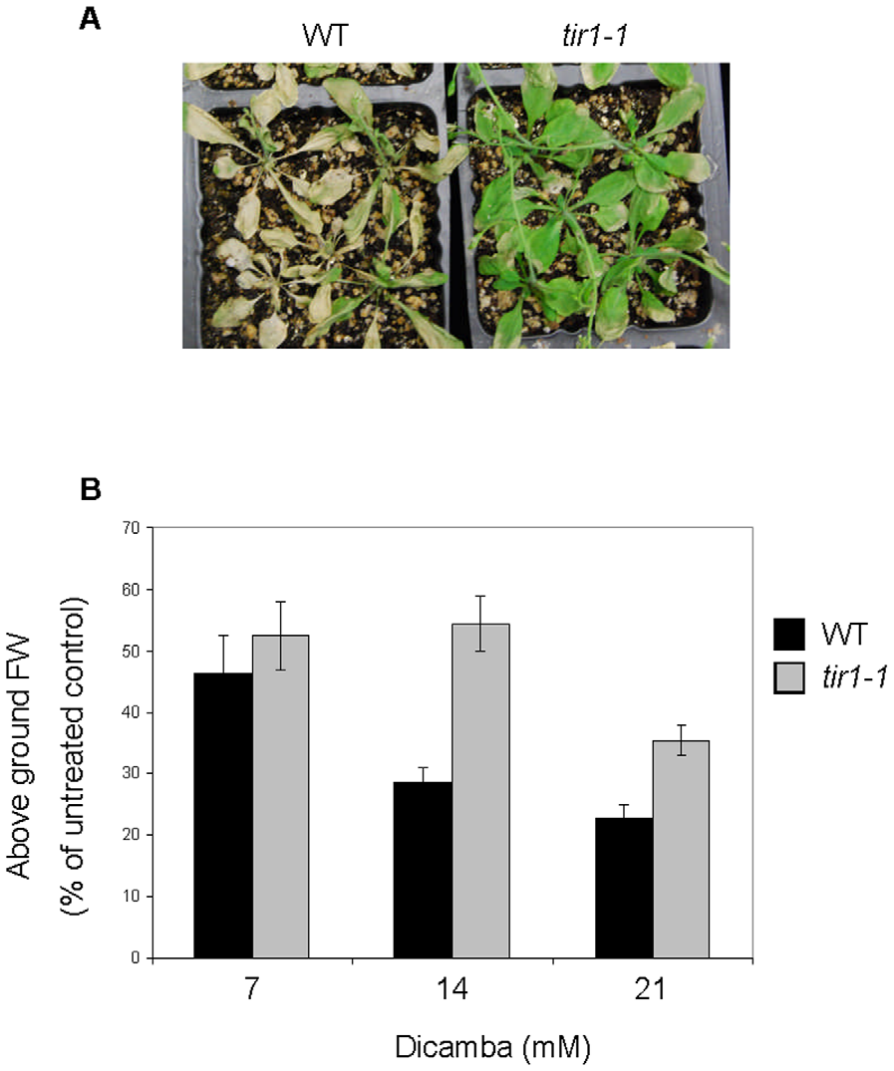
Effects of the foliar application of dicamba treatments on Arabidopsis plants. A, Representative photo showing the effects of dicamba treatment on 3 week old plants. Photo taken 3 days after a 14 mM dicamba treatment on WT and *tir1-1*. The experiment was repeated twice with similar results. B, The effects of foliar applications of dicamba on the above ground fresh weight of WT and *tir1-1* plants. Weights measured three days after treatment with 30 ml of 7, 14, and 21 mM dicamba. The change in above ground fresh weight is represented as a percentage of the weight of untreated control plants. There were 20 plants for each treatment, with the experiment repeated twice. (Error bars  =  standard error)

Since *tir1-1* was able to confer some level of resistance to Arabidopsis seedlings, we were interested to test if *tir1-1* conferred dicamba resistance to older plants. A foliar application of 7 µM dicamba on 4 week old, soil-grown WT plants caused only slight herbicidal damage. One week after treatment, the plants exhibited slightly smaller rosette diameters and no significant decreases of plant fresh weight (data not shown). Thus, although 7 µM dicamba showed effects on root growth in WT seedlings grown on agar plates, it did not have a drastic effect on older plants grown in soil. We then increased the concentration of dicamba to 7 mM, which is a concentration that falls within the normal range used to kill weeds by foliar application in agriculture [16]. When dicamba was applied to plants in soil at concentrations of 7, 14 and 21 mM, the dicamba ultimately killed WT and *tir1-1* plants, but the death was slower in *tir1-1*. We observed that 3 days post-treatment with 14 mM dicamba, the *tir1-1* plants were greener than the WT, indicating they were dying more slowly (Fig. 4A). Additionally, 3 days after treatment with 14 and 21 mM dicamba, there was significantly more above-ground fresh weight in *tir1-1* compared to the WT plants, relative to the untreated controls (Fig. 4B). Overall, our results show that *tir1-1* plants were more tolerant to dicamba treatment at both the seedling and adult stages.

### Effects of dicamba on TIR-like F-box proteins (AFBs) mutants

**Figure 5:**
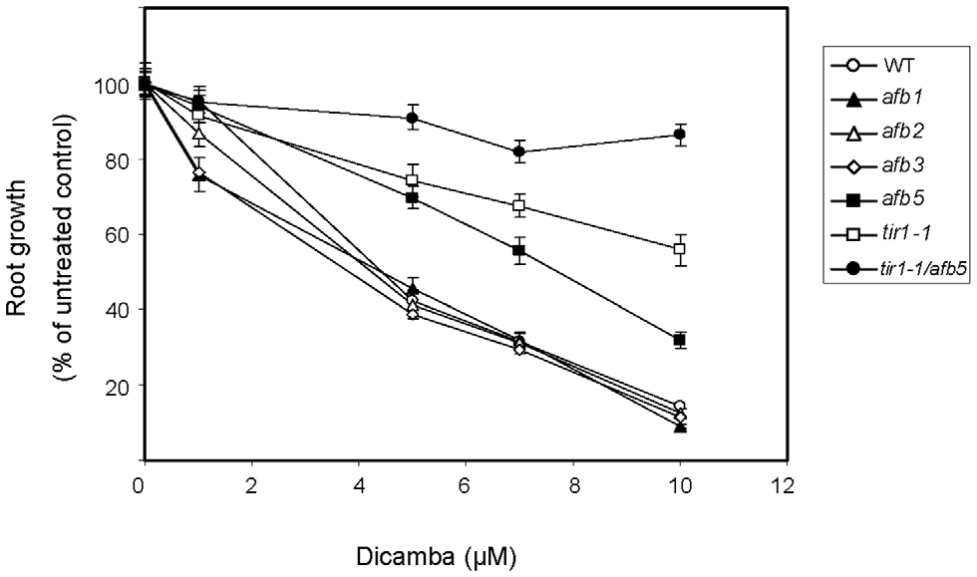
Effects of increasing dicamba concentrations on root lengths of TIR-like, F box receptor mutants. Data represents a comparison of the effects of increasing concentrations of dicamba on the root lengths of WT and TIR/AFB mutants. Roots measured after 8 days on MS-media containing 0, 1, 5, 7 or 10 µM dicamba and converted to a percentage of the average root length of the untreated control plants. Measurements were taken on at least 20 seedlings per treatment. (Error bars  =  standard error)

The fact that the *tir1-1* mutant is not completely resistant to dicamba suggests that there must be other F-box receptors mediating the dicamba response. The TIR-like F-box proteins AFB1, AFB2, and AFB3 can bind auxin, suggesting they are likely auxin receptors with overlapping roles to TIR1 [12]. More distantly related F-box proteins to TIR1 in Arabidopsis include AFB4, which is also called FBX14, and AFB5, which has been previously shown to be sensitive to 2,4-D, but resistant to the herbicide picloram [14]. To test the effect of dicamba on root growth of AFB mutants, we focused on AFB1, AFB2, AFB3, and AFB5. *afb1*, *afb2* and *afb3* were sensitive to dicamba at concentrations higher than 1 µM, similar to WT (Fig. 5). The *afb5* plants showed higher levels of resistance to the herbicide, as indicated by a significantly longer root length compared to the wildtype (Fig. 5). While *tir1-1* mutants were resistant to 0.1 µM 2,4-D, the *afb5* mutant was susceptible to this herbicide (Fig. 2A). Thus, AFB5 appears to be required to mediate responses to dicamba, but not to 2,4-D.

The *tir1-1* and *afb5* plants were crossed and the resulting homozygous double mutant was tested for dicamba resistance. The *tir1-1/afb5* plants showed enhanced resistance to dicamba at all concentrations tested (Fig 5). While concentrations of 10 µM strongly inhibited WT root growth causing an 86% reduction in root length, the double mutant had an approximately 14% decrease in root length. In addition, *tir1-1/afb5* plants showed greater resistance to dicamba than did the plants with the single mutations in *tir1-1* or *afb5*, suggesting an additive effect of the mutations on dicamba resistance and that both contribute to the dicamba response.

### Gene expression analysis in the *tir1-1* and *afb5* mutant backgrounds

We have shown that TIR1 and AFB5 are required, at least in part, for dicamba mediated effects. In order to determine if the *tir1-1* and/or *afb5* mutation affects dicamba-regulated, downstream gene expression, we analysed changes in transcript levels after a 10 hour, 7 mM dicamba treatment in the WT, *tir1-1*, *afb5*, *and tir/afb5* backgrounds using qRT-PCR for the following auxin-regulated genes: *IAA1*, *IAA5*, *GH3.3*, (auxin- mediated responses), *NCED3*, (ABA biosynthesis), *ABF4* (ABA signalling), and *GSTF8* (stress responses).

**Figure 6:**
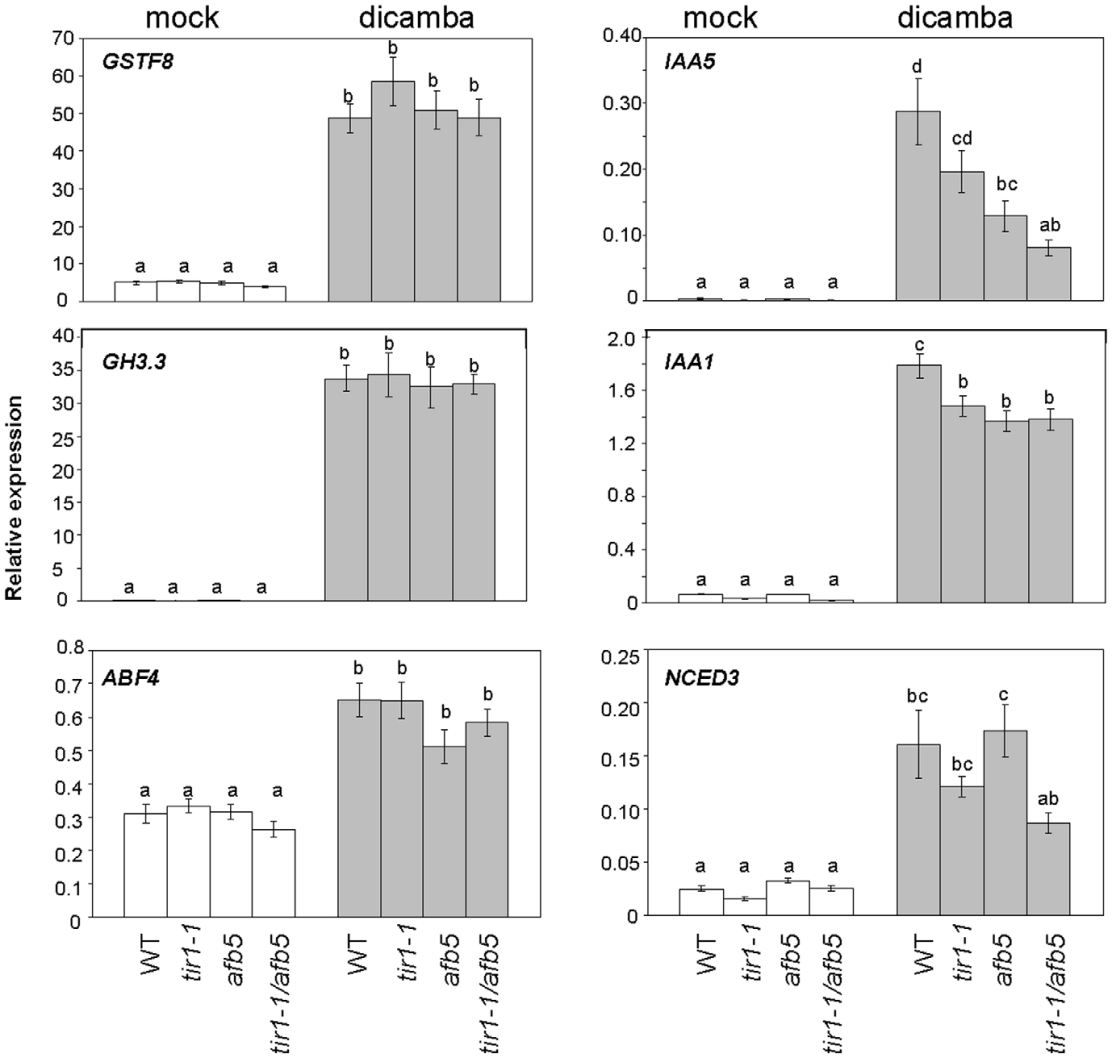
Consequences of *tir1-1*, *afb5*, and *tir1-1/afb5* on the dicamba-induced expression of select genes. Transcript expression was monitored by qRT-PCR in Col-0, *tir1-1*, *afb5*, and *tir1-1/afb5* plants after a 10 hour, 7 mM dicamba treatment. The following genes were analysed: *GSTF8* (At2g47730), *GH3.3* (At2g23170), *ABF4* (At3g19290), *IAA5* (At1g15580), *IAA1* (At4g14560), and *NCED3* (At3g14440). The graphs show the average relative gene expression from two independent experiments, each containing three biological replicates. (Error bars  =  standard error). The different letters indicate differences by Least Square Means Tukey HSD (p<0.05). Levels not connected by the same letter are significantly different.

First, we looked at the effects of dicamba on WT plants. The relative expression for the 6 genes described above was significantly increased in the WT-dicamba treated plants compared to the WT mock-treated controls (Fig. 6). This data is consistent with the changes in gene expression observed in the microarray experiment.

Next, the effects of dicamba on relative gene expression in the *tir1-1*, *afb5*, and *tir1-1/afb5* backgrounds were analysed. We measured relative gene expression for these 6 genes in the mutant dicamba-treated plants and compared it to relative gene expression in the WT dicamba-treated plants (Fig. 6). Transcripts for the stress-responsive *GSTF8*, auxin-conjugating *GH3.3*, and the ABA-responsive *ABF4* were up-regulated in all the dicamba-treated mutant plants similar to WT (Fig. 6). However, for the auxin responsive gene *IAA1*, the level of relative dicamba-induced gene expression in the three mutants (*tir1-1*, *afb5*, and *tir1-1/afb5*) was significantly less than in WT (Fig. 6). For *IAA5* there was less dicamba-induced transcription in *afb5* and *tir1-1/afb5* compared to WT. In addition, the double mutant showed a trend towards decreased gene expression of the ABA-biosynthetic gene *NCED3* (Fig. 6). Overall, the *tir1-1* and/or *afb5* mutations were having an effect on the induction of some but not all of the dicamba-induced genes, suggesting both complexity and redundancy of the downstream gene regulation following application of this herbicide.

## Discussion

In this report we have provided evidence for chemical specificity of plant responses to auxinic herbicides and a gene expression study for dicamba-treated plants. Herbicide resistant weed populations, especially to the commonly used herbicide glyphosate, are a growing problem [43], [44]. Approximately 90% of all transgenic crops worldwide are glyphosate resistant; thus, glyphosate-resistant weeds pose a serious threat to agricultural systems [16], [45]. Synthetic auxins such as dicamba are an attractive alternative to glyphosate. They are effective, non-toxic herbicides that despite over 60 years of broad use, have not resulted in significant issues with the emergence of resistant weeds, probably owing to their multiple sites of action [46]. However, with farmers increasing their usage of dicamba because of resistant weeds to other herbicides, including glyphosate, triazine, and acetohydroxyacid synthase-inhibiting herbicides [47], and because of the future introduction of dicamba resistant transgenic crops, the selection pressure for dicamba resistance in weeds will be amplified. In fact, dicamba resistant weeds have already been reported, for example in the broadleaf weed Kochia [18], [48] and in wild mustard *Brassica kaber* [19], underscoring the importance of investigating dicamba's mode of action at the genetic level. The transcript analysis reported here will be helpful in further understanding incidents of spontaneous dicamba resistance arising in weeds.

### Transcriptional analysis of dicamba-treated plants suggests prolonged stress and phytohormone responses

Our analysis of dicamba-regulated genes using a whole genome Arabidopsis array provided insight into the molecular mechanisms underlying dicamba action. Although microarrays have been used to examine plant transcriptional responses after treatment with various concentrations of IAA or synthetic auxins, such as 2,4-D, these investigations focused primarily on early responses, shortly after treatment[33], [34], [49], [50], [51], [52], [53]. Moreover, while a previous report by Pufky and associates had utilized a microarray of dicamba treated plants, it did not contain a detailed examination of dicamba-regulated genes, and instead the array data was used to compare overall expression patterns between different classes of auxinic herbicides 20 minutes after their application [32]. To date there are no reports looking at the prolonged effects on plant transcript levels after a long (10 hr) auxinic herbicide treatment. As a result, our work can give insights on the plant responses regulated by synthetic auxins that occur subsequent to their initial exposure and provide new opportunities for auxinic herbicide research, such as identifying novel genes for diagnosing off-target herbicidal injury [2]. Such genes could help farmers differentiate between the effects of accidental herbicide exposure from nearby, sprayed fields and possible disease outbreaks.

In our analysis, there were over 550 genes up-regulated by 10 hour treatment with dicamba (Table S1). The current model describing the mode of action of auxinic herbicides proposes that massive doses of auxin lead to enhanced levels of ethylene and ABA, resulting in plant cell death [3], [4]. Overall, our microarray analysis provides further molecular support for this model. For example, three ACC synthases were induced by dicamba. This is consistent with previous work showing that auxinic herbicides can stimulate ACC synthase activity and increase endogenous levels of ethylene in sensitive plants [4], [6], [7], [54]. Following the auxin-triggered induction of ACC synthase is the overproduction of ABA [6]. We observed an up-regulation of ABA biosynthetic genes, with dicamba up-regulating transcription of both *NCED3* and *NCED5*, suggesting that dicamba-treated plants are experiencing *de novo* ABA biosynthesis.

Natural auxins are rapidly degraded by the plant, but auxinic herbicides are not readily metabolized by sensitive plants, and this probably leads to lasting effects on gene expression. By using a promoter::reporter system, we found that *GSTF8* promoter activity was strongest approximately 10 hours after dicamba treatment. *GSTF8* is a marker for plant stress responses [23], [24], [55], [56], [57], [58] and its induction at 10 hrs may indicate the plant is experiencing ongoing stress. Normally, as the cellular concentrations of auxins decline, the transcription of auxin responsive genes is also downregulated [21]. However, our gene expression analysis showed strong induction of Aux/IAA genes and other auxin responsive genes at 10 hours post dicamba treatment, suggesting a prolonged presence of dicamba within the cell, perhaps because dicamba is not readily inactivated or detoxified by the cell. To help deal with high levels of auxin, GH3 proteins are usually induced as they can conjugate natural auxin for degradation. *GH3* genes were induced by dicamba, suggesting that the plant was attempting to remove excess auxin. However dicamba is not a substrate of the GH3 enzymes [59], which may be why plants are unable to cope with this herbicide [2]. We also observed strong induction of *NCED* and ACC synthase genes 10 hours after dicamba treatment, indicating that dicamba is triggering on-going, ethylene and ABA biosynthesis in the plant.

### Dicamba mediates its affect independently of AXR4, but relies at least in part on F-box auxin receptors TIR1-1 and AFB5

For the induction of auxin specific genes, auxin is first perceived by the plant through its TIR1/AFB proteins and requires transport both in and out of the cell via auxin import/export proteins. By testing dicamba and 2,4-D sensitivity in the auxin insensitive Arabidopsis mutants affected in either auxin transport (*axr4-2*) or auxin perception (*tir1-1*), we showed that while both *axr4-2* and *tir1-1* were resistant to 2,4-D, only *tir1-1* was resistant to dicamba. The *axr4-2* and the *tir1-1* mutants affect different components of the auxin-mediated signalling pathway. The auxin-insensitive mutant *axr4* is defective in auxin responses due to the mislocalization of AUX1, an auxin influx carrier [60]. In *axr4*, AUX1 is not correctly targeted to the plasma membrane, and as a result, auxin transport into the cell is disrupted. Previous reports have shown that membrane-localized AUX1 is required for the uptake of 2,4-D by the cell [61]. 1-NAA but not 2,4-D can successfully enter the cells without the need for an auxin influx carrier [62]. Because 1-NAA is more hydrophobic than 2,4-D or IAA, it may more freely diffuse into the cells [62]. *axr4* is susceptible to dicamba, indicating that dicamba may also permeate the cells without requiring an auxin carrier for cellular uptake. Alternatively, dicamba may rely on a different auxin influx carrier to enter the cell.

The TIR1 protein is required for the degradation of Aux/IAA transcriptional repressors, and a mutation in TIR1 causes diminished auxin responses. TIR1 contains a binding pocket that can bind IAA and can also bind two auxin analogs, 2,4-D and 1-napthalenacetic acid (1-NAA) [10], [15], [63]. The TIR1 binding pocket had the highest affinity for IAA, and although 2,4-D could bind to TIR1, it was bound with weaker affinity [64]. The differences in affinity between synthetic and natural auxins were linked to the size of their aromatic ring structures, with 2,4-D having a smaller ring size than IAA [63]. The fact that *tir1-1* plants were partially resistant to dicamba suggests that TIR1 can bind directly to dicamba with the binding affinity influenced by the structure and size of dicamba. Computer modelling also has suggested that dicamba can fit into the auxin binding site of TIR1 [3].

The TIR1 homologs AFB1, AFB2, and AFB3 are closely related to TIR1, with 61-72% amino acid identity. Plants deficient in these proteins are more sensitive to 2,4-D than *tir1-1* [13]. Earlier studies on understanding the roles of the AFB proteins showed that TIR1 and AFB2 made the largest contribution to the auxin response in roots; however, it should be noted that AFB5 was absent from this work [13]. The AFB5 protein is more distantly related to TIR1 with only 46% amino acid identity, and our data showed that while the *afb5* mutant was sensitive to 2,4-D, it was relatively resistant to dicamba treatment. Further evidence that AFB5 contributes to the dicamba response comes from the double, *tir1-1/afb5* mutant, which showed stronger resistance to dicamba than either the *tir1-1* or *afb5* single mutants. Previously, the *afb5* mutant was reported to confer specific resistance to picolinate auxins, but no resistance to IAA, 2,4-D and dicamba [14]. It is not apparent why this study did not find similar dicamba resistance, but our results clearly show enhanced resistance of *afb5* to dicamba treatment and that AFB5 is contributing to the dicamba response.

Since the *tir1-1* mutant showed resistance to both dicamba and 2,4-D, TIR1 may be mediating the overlapping, downstream responses to herbicides. However, *TIR1-1* is not involved in regulating all genes after dicamba treatment, as highlighted by the gene expression studies (Fig. 5). Some auxin-regulated gene expression may be regulated through signalling pathways other than TIR1, and could involve AFB5. Both SCF^AFB5^ and SCF^TIR1^ may be contributing to distinct dicamba responses, with perhaps auxin chemistry regulating the specific contribution of each SCF. Interestingly, mutations in *TIR1-1* and/or *AFB5* significantly affected gene expression of *IAA1.* However, the fact that for most of the genes tested, expression was not altered in the mutant *tir1-1* and/or *afb5* backgrounds suggests that either there is a different mechanism for mediating the dicamba-induced expression of these genes that does not involved TIR1 or AFB5, or that the other signalling F-box receptors play a role.

Although analysis of the *tir1-1/afb5* double mutant (this study), as well triple and quadruple *tir1/afb1*,*2*,*3* mutants [13] has suggested the AFB proteins have overlapping functions, unravelling the contribution of each family member to the complex auxin responses is still a work in progress. Further clarification of the role of the TIR homologs (AFB1, AFB2, and AFB3) has come from a recent study which reported that the function of the different TIR/AFB proteins in the auxin response could be attributed to their relative expression, which is under the control of post-transcriptional regulation, or related to their binding to different Aux/IAA substrates [13]. One theory for the large number of TIR1 homologs is that although IAA is the major form of auxin, there may be other structurally different forms of natural auxin playing roles in developmental processes, with AFB proteins providing differing specificity for these compounds [8]. The specificity of AFB5 for picloram and dicamba supports this model.

Overall, the detailed dicamba-regulated gene expression studies support the hypothesis that auxinic herbicides work in large part through manipulating the plants phytohormone responses, specifically causing large increases in ethylene and ABA levels. By minimising the manipulation of the phytohormones in response to these herbicides, it may be possible to develop additional means of auxinic herbicide resistance. Although both 2,4-D [33], [34] and dicamba (this work) induce similar phytohormone responses in the plant, auxin insensitive mutants showed differential responses to these herbicides. Our mutant work suggests that variation in synthetic auxin sensitivity is regulated by two components. The first component is cellular uptake of the synthetic auxins. As illustrated by the differential sensitivity of *axr4-2* to 2,4-D and dicamba, this component may influence the effectiveness of certain herbicides. As a result, auxin transport could be targeted for manipulation to provide additional specificity for synthetic auxins. The second component of selectivity in synthetic auxins was conferred by F-box receptors. We have demonstrated that plants containing mutations in AFB5 had specific resistance to dicamba, and that this resistance was additive in the *tir1-1/afb5* double mutant, indicating both contribute to dicamba resistance. In this context, it may be possible that other members of the TIR1/AFB family have distinct auxin specificities that can be modified to develop new herbicide resistances. For example, it may be possible to use TILLING (Targeting Induced Local Lesions in Genomes) to identify mutants in specific auxin receptors that contribute to increased herbicide resistance in crop plants. This approach would offer a non-GM approach to the engineering of novel auxinic herbicide resistances.

## Supporting Information

Table S1 **Excel file of all 551 probe identifiers induced by dicamba 3 fold or more on the Arabidopsis Affymetrix array.** The ACC number, Affymetrix probe ID, Log_2_FC, and gene description are included for each gene.
(XLS)

Table S2 **Excel file of all 379 probe identifiers repressed by dicamba 3 fold or more on the Arabidopsis Affymetrix array.** The ACC number, Affymetrix probe ID, Log_2_FC, and gene description are included for each gene.
(XLS)

Table S3 **Primers used in qRT-PCR.**
(DOC)
