# Supplementary material for: Mutant Analysis in Arabidopsis Provides Insight into the Molecular Mode of Action of the Auxinic Herbicide Dicamba
Source: PLoS One. 2011 Mar 8;6(3):e17245. doi: 10.1371/journal.pone.0017245 (PMC3050828; doi:10.1371/journal.pone.0017245)
Supplement: Table S3 — Primers used in qRT-PCR. (DOC) [file pone.0017245.s003.doc]

Supplemental Table S3

Primers used in qRT-PCR
